# Synthesis and Antibacterial Activities of Novel 2,5-Diphenylindolo[2,3-*e*] Pyrazolo[1',5':3",4"]pyrimido[2",1"-*c*][1,2,4]triazines

**DOI:** 10.3390/molecules161210387

**Published:** 2011-12-15

**Authors:** Kamal F.M. Atta, Omaima O.M. Farahat, Somaya M. Ghobashy, Mohamed G. Marei

**Affiliations:** Department of Chemistry, Faculty of Science, Alexandria University, Ibrahimia P.O. Box 426, Alexandria 21321, Egypt

**Keywords:** pyrazolopyrimidine, isatin, hydrazone, indolopyrazolopyrimidotriazine, antibacterial activity

## Abstract

The formation of (*E*)-3-{2-(2,5-diphenylpyrazolo[1,5-*c*]pyrimidin-7-yl)hydrazono}indolin-2-ones **3** has been achieved by condensation of equimolar amounts of 7-hydrazino-2,5-diphenylpyrazolo[1,5-*c*]pyrimidine (**1**) and isatin (or isatin derivatives) **2**at room temperature. The (*E*)-products could be isomerized into corresponding the (*Z*)-**3** isomers. Reactions of the latter fused heterocyclic hydrazones towards different electro-philic reagents yielded the corresponding 3-substituted derivatives **4–7**. Dehydrative cyclisation of the hydrazones **3** using phosphorus oxychloride afforded the 2,5-diphenyl- indolo[2,3-*e*]pyrazolo[1',5':3",4"]pyrimido[2",1"-*c*][1,2,4] triazines **13**. The polyfused heterocyclic ring system **13** underwent electrophilic substitution reactions at position 4 rather than at position 3. The 3-bromo isomer of **17** was prepared by a sequence of reactions starting from 2,5-diphenylpyrazolo[1,5-*c*]pyrimidine-7(6*H*)-thione (**11**). The orientation of the electrophilic attack was supported by spectroscopic and chemical evidence. Some of the synthesized compounds were found to possess slight to moderate activity against the microorganisms *Bacillus subtilis*, *Micrococcus luteus*, *Staphylococcus aureus*, *Escherichia coli * and *Pseudomonas aeruginosa*.

## 1. Introduction

Pyrimidines and fused pyrimidines, being an integral part of DNA and RNA, play an essential role in several biological processes. They also have considerable chemical and pharmacological importance; particularly, as nucleoside antibiotics, antibacterial, cardiovascular as well as agrochemical and veterinary products [1,2,3,4,5,6,7,8,9]. Various pyrimidine derivatives showed analgesic, antiarrhythmic, and anticancer activities [10,11,12], as well as anti-inflammatory, antiparkinsonian, and androgenic anabolic activities [13,14,15,16,17,18].

Isatin is known to be a colorimetric reagent for the amino acid proline, forming blue derivatives [19]. This property has been exploited for the determination of the level of this amino acid in pollens [20] or for the detection of polymer bound compounds possessing proline residues [21]. It has also been used in a colorimetric screening test for human serum hyperprolinaemia [22], in a colorimetric assay of HIV-1 proteinase [23] and for the estimation of the age of bones in crime investigation [24]. In a similar manner, isatin-3-hydrazone has been studied for the colorimetric determination of steroids [25,26].

Encouraged by the above observations and in continuation of our work for the syntheses of heterocyclic compounds from hydrazino heterocycles [27,28,29,30,31,32], a new series of 3-{2-(2,5-diphenyl- pyrazolo[1,5-*c*]pyrimidin-7-yl)hydrazono}indolin-2-ones and 2,5-diphenylindolo[2,3-*e*]pyrazolo- [1',5':3",4"]pyrimido[2",1"-*c*][1,2,4]triazines were synthesized, with a view to explore the possibility of achieving better biological activities.

## 2. Results and Discussion

**Scheme 1:**
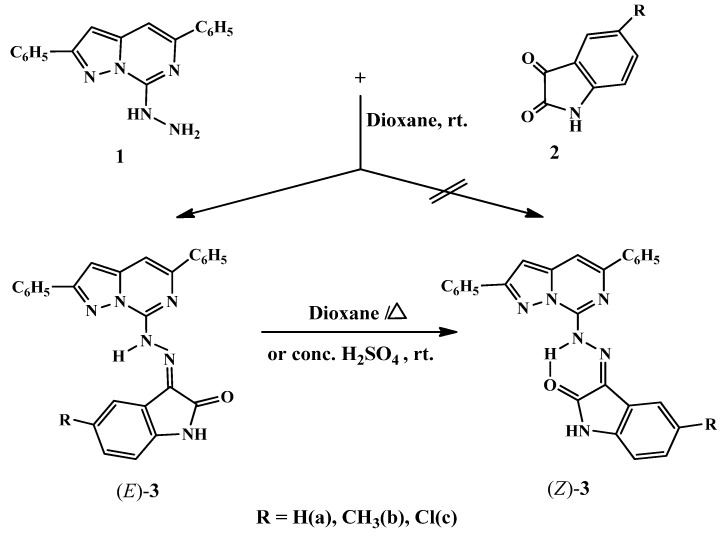
Synthesis of 3-{2-(2,5-diphenylpyrazolo[1,5-*c*]pyrimidin-7-yl)hydrazono}indolin-2-ones **3**.

The theoretical existence of geometric isomers of 3-{2-(2,5-diphenylpyrazolo[1,5-*c*]pyrimidin-7-yl)hydrazono}indolin-2-ones (*E* and *Z*)-**3** had been predicted for the condensation of 7-hydrazino-2,5-diphenylpyrazolo[1,5-*c*]pyrimidine (**1**), which was readily obtained by sequence of reactions starting from ethyl phenylpropiolate [33,34], with isatin (or isatin derivatives) **2** (Scheme 1). But by stirring equimolar amounts of **1** with **2** at room temperature the reaction yielded only the kinetically more stable geometrical isomer (*E*)-**3a–c**, which upon heating in dioxane or stirring with conc. H_2_SO_4_ at room temperature underwent isomerisation to give the thermodynamically more stable isomer (*Z*)-**3a–c** showing a possibility of hydrogen bond formation. The structure and configuration of the pyrazolopyrimidinoindolinonehydrazones (*E* and *Z*)-**3** were fully differentiated by studying their spectra, which included IR, ^1^H-NMR and MS. The IR spectra showed characteristic five membered ring amide carbonyl absorption bands at 1684–1710 and 1684–1692 cm^−1^, in addition to the NH absorption band in the range 3459–3479 and 3451–3467 cm^−1^, respectively.

**Figure 1:**
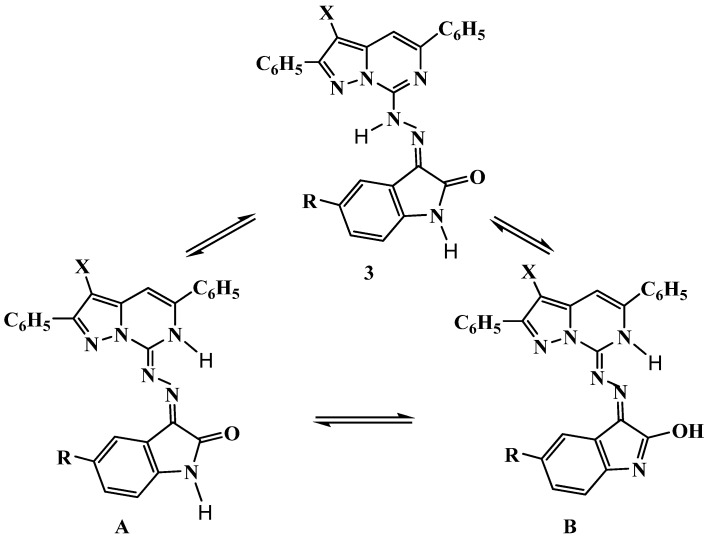
Isomerisation of (*E*)-3-{2-(2,5-diphenylpyrazolo[1,5-*c*]pyrimidin-7-yl)hydrazono}indolin-2-ones (*E*)-**3**.

The ^1^H-NMR spectra of (*E*)-**3a–c** revealed, besides the aromatic protons as a multiplet at δ_H_ 7.37–8.04, two doublets at δ_H_ 8.07–8.11 and at δ_H_ 8.16–8.23, as well as other characteristic singlets at δ_H_ 6.85–7.23 for the H-3 pyrazole ring proton and at δ_H_ 7.54–7.92 for the H-4 pyrimidine ring proton. The assignment of the higher field signal for the H-3 pyrazole ring proton and the lower field signal for H-4 pyrimidine ring protons is supported by the data reported for 2,5-diarylpyrazolo[1,5-*c*]pyrimidine-7(6*H*)-thiones [20]. Moreover, the spectra of (*E*)-**3a–c** exhibited exchangeable singlets at δ_H_ 10.43–10.80 and at δ_H_ 14.19–14.22 which are attributed to the NH of hydrazone conformer **3** and NH of pyrimidine conformers **A** or **B**. The intensity of both singlets is equivalent to one proton. The spectra also showed an exchangeable proton as two singlets equivalent to one proton at δ_H_ 11.03–11.36 and at δ_H_ 11.15–12.17 which were ascribed to the NH conformer **3** and OH conformer **B** of indole ring [35]. Furthermore, the spectrum of (*E*)-**3b** showed a singlet at δ_H_ 2.33 for the CH_3_ group. The previous data indicates that pyrazolopyrimidinoindolinonehydrazones (*E*)-**3a–c** exist as a mixture of the toutomers **3**, **A** and **B** (Figure 1).

The ^1^H-NMR spectra of (*Z*)-**3a–c** showed, besides the aromatic protons as a multiplet at δ_H_ 7.37–7.71, two doublets at δ_H_ 8.11–8.13 and at δ_H_ 8.22–8.27, as well as other characteristic singlets at δ_H_ 7.18–7.22 for the H-3 pyrazole ring proton and at δ_H_ 7.88–7.95 for the H-4 pyrimidine ring proton. The spectra of (*Z*)-**3a–c** also exhibited an exchangeable NH proton at δ_H_ 11.14–11.37 which was ascribed to the indole ring [35] and at δ_H_ 14.21–14.23 for the chelated NH hydrazone residue. On the other hand, the spectra of (*Z*)-**3b** showed a singlet at δ_H_ 2.33 for the CH_3_ group. The above ^1^H-NMR spectral data showed only a single conformer for the structure of the hydrazone (*Z*)-**3**.

Further conformation for the structure of both (*E* and *Z*)-pyrazolopyrimidinoindolinonehydrazones was obtained from their mass spectral data, where both isomers showed similar molecular ion peaks at *m/z* 430, 444 and 464, in addition to base peaks at *m/z* 77, 339 and 359 for derivatives **a–c**, respectively, in addition to the same fragments with similar or almost similar intensities.

**Scheme 2:**
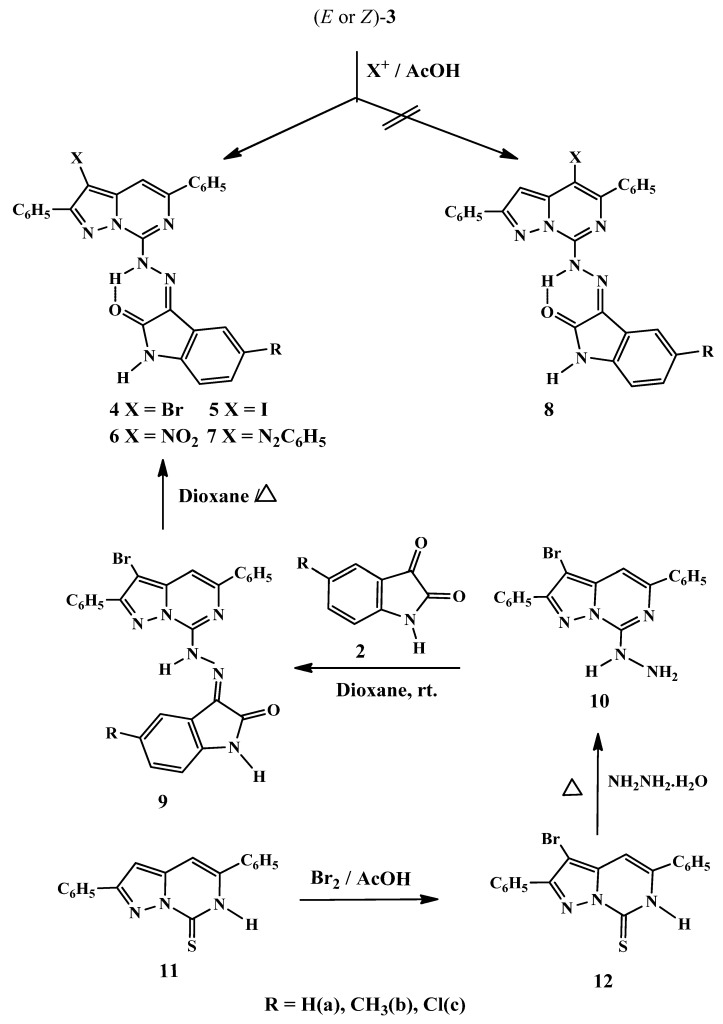
Electrophilic substitution reactions of 3-{2-(2,5-diphenylpyrazolo[1,5-*c*]pyrimidin-7-yl)hydrazono}indolin-2-ones (*E* or *Z*)-**3**.

In the present investigation the electrophilic substitution reactions of the geometrical isomers pyrazolopyrimidinoindolinonehydrazones (*E* or *Z*)-**3a–c** were studied in the hope that introduction of such substituents might enhance their biological properties, as well as, to study the more reactive position for the electrophilic attack on such fused heterocyclic rings (Scheme 2). Thus, bromination of (*E* or *Z*)-**3a–c** with bromine in glacial acetic acid, as well as, iodination with iodine monochloride in the same solvent yielded the respective monosubstituted (*Z*)-isomers **4** and **5**, since the (*E*)-**3a–c** isomers were proved to convert into the respective (*Z*)-conformers in acidic medium.

Moreover, reaction of (*E* or *Z*)-**3a–c** with nitric and sulfuric acids in glacial acetic acid and with benzenediazonium chloride in the presence of sodium hydroxide afforded the (*Z*)-3-nitro and 3-phenyldiazenyl derivatives **6** and **7**, respectively.

The structures of the 3-substituted derivatives **4–7** were confirmed by their spectral data. The ^1^H-NMR spectra of **4a–c** and **5a–c** showed the absence of the H-3 pyrazole ring proton signals and the presence of the H-4 pyrimidine ring proton as singlet at δ_H_ 7.61–7.93 ppm.

The structures were further confirmed chemically by preparing the isomeric 3-bromo derivatives **9a–c** through the bromination of 2,5-diphenylpyrazolo[1,5-*c*]pyrimidine-7(6*H*)-thione **11** with bromine in acetic acid rather than bromine in chloroform which gave the respective 3-bromo derivative **12** [30,33,34] (Scheme 2). Refluxing of **12** with hydrazine hydrate in ethanolic solution afforded the respective hydrazino derivatives **10**, which upon stirring with isatin (or isatin derivatives) **2** at room temperature yielded the corresponding (*E*)-hydrazono derivatives **9a–c**, which underwent isomerisation upon heating in dioxane to give the geometrical isomers (*Z*)-**4a–c**. The isomeric structure of hydrazones **4** and **9** are different in shape under the microscope and by TLC (R_f_ = 0.65, 0.77, 0.75 and 0.27, 0.43, 0.42), respectively, in addition to their mp. 288–290, 320–322, 304–306, 312–314, 300–302, 308–310 °C, respectively.

The ^1^H-NMR spectrum of (*E*)-**9a** showed, besides the aromatic protons as a multiplet at δ_H_ 7.87–8.05, two doublets at δ_H_ 8.08 and at δ_H_ 8.25, as well as another characteristic singlet at δ_H_ 7.74 for the H-4 pyrimidine ring proton. Moreover, the spectrum of **9a** exhibited exchangeable singlets at δ_H_ 10.92 and at δ_H_ 14.16 which are attributed to the NH of the hydrazone conformer **3** and the NH of the pyrimidine conformers **A** or **B**, respectively. The intensity of both singlets is equivalent to one proton. The spectrum also revealed an exchangeable proton as two singlets equivalent to one proton at δ_H_ 11.21 and at δ_H_ 11.29 which ascribed to the NH conformer **3** and OH conformer **B** of the indole ring [46] (Figure 1).

**Scheme 3:**
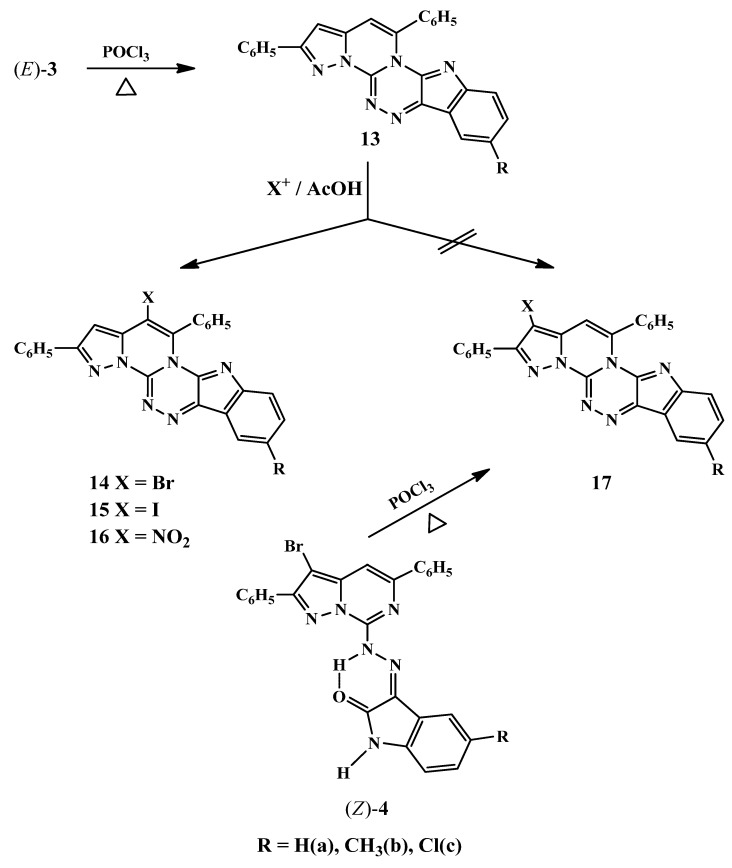
Annulation of 2,5-diphenylindolo[2,3-*e*]pyrazolo[1',5':3",4"]pyrimido[2",1"-*c*][1,2,4]triazines.

The high point in the present investigation is the cyclization of the (*E*)-pyrazolopyrimido-indolinonehydrazones **3a–c** forming novel polycyclic rings with six heteroatoms containing two bridged nitrogens. Thus, heating of (*E*)-**3a–c** with phosphorus oxychloride afforded the corresponding target 2,5-diphenylindolo[2,3-*e*]pyrazolo[1',5':3":4"]pyrimido[2",1"-*c*][1,2,4]-triazines **13a–c** (Scheme 3). The structure of the indolopyrazolopyrimidotriazines was fully established from their spectral data analysis, which included IR, ^1^H-NMR and MS spectra. The ^1^H-NMR spectra of the **13a**,**b** revealed, besides the aromatic protons as a multiplet at δ_H_ 7.33–7.55, two doublets at δ_H_ 8.01, 8.13 and at δ_H_ 8.13, 8.20, as well as other characteristic singlets at δ_H_ 7.42, 7.53 for the H-3 pyrazole ring proton and at δ_H_ 7.55, 7.91 for the H-4 pyrimidine ring proton. On the other hand, the spectrum of **13b** exhibited a singlet at δ_H_ 2.44 for the CH_3_ group.

The mass spectra of the heterocyclic compounds **13a–c** confirmed the dehydrative cyclisation of the respective hydrazones showing their molecular ion peaks at *m/z* 412, 426 and 446, respectively, compared to that of the starting reactants at *m/z* 430, 444 and 464, respectively.

The novel fused indolopyrazolopyrimidotriazines **13a–c** appeared to be attractive intermediates for the synthesis of a number of substituted derivatives via reaction with some representative electrophilic reagents, and to the best of our knowledge, no reports on the electrophilic substitution reactions of the indolopyrazolopyrimidotriazine ring system have been published. We are interested in investigating the reactivity at either the C-3 or C-4 position in such heterocyclic rings. Thus, bromination of **13a–c** with bromine, as well as, iodination with iodine monochloride gave the respective 4-bromo **14a–c** and 4-iodo **15a–c** derivatives, respectively. Moreover, nitration of **13a–c** with nitric and sulfuric acids in glacial acetic acid afforded the respective 4-nitro derivatives **16a–c**. The structures of the 4-substituted derivatives **14–16** were confirmed by studying their ^1^H-NMR spectra, which showed the disappearance of the H-4 pyrimidine ring proton signals and the appearance of the H-3 pyrazole ring proton signals at δ_H_ 7.28–7.47.

Furthermore, the structures of **14–16** were confirmed chemically by synthesizing the 3-substituted isomeric derivatives **17**. Thus, refluxing of (*Z*)-**4a–c** with phosphorus oxychloride led to the formation of the respective isomeric 3-bromo-derivatives of the fused triazines** 17a–c**. The two isomeric bromo derivatives **14** and **17** were found to be completely different (TLC, mp and mixed mp, IR, ^1^H-NMR and MS spectra). The ^1^H-NMR spectra of **17a**,**b** showed the absence of the H-3 pyrazole ring proton signals and the presence of the H-4 pyrimidine ring proton signals at δ_H_ 7.69, 7.94, respectively.

## 3. Experimental

### 3.1. General

Melting points were determined on a Kofler block and are uncorrected. Elemental analyses were carried out in the Microanalytical Laboratory of the Faculty of Science, Cairo University. The IR spectra of compounds were recorded on a Fourier Transform infrared 8400 spectrophotometer [Bruker Tensor 37] using potassium bromide pellets and frequencies are reported in cm^−1^. The ^1^H-NMR spectra were recorded on a JEOL JNM ECA 500 MHZ instrument and chemical shifts δ_H_ are in ppm relative to tetramethylsilane used as internal standard. Mass spectra were recorded at 70 ev with a GCMS-QP 1000 EX spectrometer. Reactions were routinely followed by thin layer chromatography (TLC) Merck Kiesel gel; 60-F254 precoated plastic plates. The spots were detected by iodine. 5-Aryl-7-hydrazino-2-phenylpyrazolo[1,5-*c*]pyrimidines **1** and **10** were prepared from the respective acetylenic β-diketones as described earlier [30,33,34].

### 3.2. Synthesis of Compounds

#### 3.2.1. (E)-3-{2-(2,5-Diphenylpyrazolo[1,5-c]pyrimidin-7-yl)hydrazono}indolin-2-ones **3a–c**

A solution of 2,5-diphenyl-7-hydrazinopyrazolo[1,5-*c*]pyrimidine [30,33] (**1**, 0.30 g, 0.0010 mol) in dioxane (10 mL) was stirred with isatin (or isatin derivatives) (**2**, 0.0015 mol) for 24 hours at room temperature. The products that separated out as orange needles were filtered off, washed with methanol and dried.

*(E)-3-{2-(2,5-Diphenylpyrazolo[1,5-c]pyrimidin-7-yl)hydrazono}indolin-2-one *(**3a**). Yield 70%; m.p. 332–334 °C; R_f_ 0.18 (3:1 benzene-EtOAc); IR (cm^−1^): 3479 (NH), 1700 (indole ring C=O), 1619 (pyrazole ring C=N), 1565 (pyrimidine ring C=N) and 1452 (pyrimidine ring C=C); ^1^H-NMR (DMSO-*d_6_*, δ_H_, ppm): 6.85 (s, 1H, pyrazole-H), 7.37–8.04 (m, 10H, aromatic-H), 7.54 (s, 1H, pyrimidine-H), 8.07 (d, 2H, aromatic-H), 8.16 (d, 2H, aromatic-H), 11.09, 11.23 (s, 1H, exchangeable NH, OH) and 10.65, 14.20 (s, 1H, exchangeable NH); MS, *m/z* (%): 430 (7, M^+^), 402 (1, M^+^-N_2_), 325 (17, M^+^-C_7_H_7_N), 248 (1, M^+^-C_13_H_10_O), 234 (1, M^+^-C_13_H_12_N_2_), 194 (1, M^+^-C_14_H_10_N_3_O^−^), 165 (2, M^+^-C_15_H_13_N_4_O), 139 (4, M^+^-C_16_H_13_N_5_O), 132 (4, M^+^-C_19_H_12_N_3_O), 88 (3, M^+^-C_21_H_18_N_4_O), 77 (100, M^+^-C_20_H_13_N_6_O) and 62 (8, M^+^-C_22_H_18_N_5_O); Anal. Calc. for C_26_H_18_N_6_O (430.46): C, 72.55; H, 4.21; N, 19.52%, found: C, 72.48; H, 4.17; N, 19.47%.

*(E)-3-{2-(2,5-Diphenylpyrazolo[1,5-c]pyrimidin-7-yl)hydrazono}-5-methylindolin-2-one* (**3b**). Yield 88%; m.p. 330–332 °C; R_f_ 0.28 (3:1 benzene-EtOAc); IR (cm^−1^): 3459 (NH), 1684 (indole ring C=O), 1631 (pyrazole ring C=N), 1547 (pyrimidine ring C=N) and 1460 (pyrimidine ring C=C); ^1^H-NMR (DMSO-*d_6_*, δ_H_, ppm): 2.33 (s, 3H, CH_3_), 7.18 (d, 1H aromatic-H) 7.23 (s, 1H, pyrazole-H), 7.44–7.56 (m, 8H, aromatic-H), 7.89 (s, 1H, pyrimidine-H), 8.11 (d, 1H, aromatic-H), 8.18 (t, 2H, aromatic-H), 8.22 (d, 1H, aromatic-H), 11.03, 11.15 (s, 1H, exchangeable NH, OH) and 10.80, 14.22 (s, 1H, exchangeable NH); MS, *m/z* (%): 444 (41, M^+^ ), 416 (36, M^+^-N_2_), 339 (100, M^+^-C_7_H_7_N), 262 (1, M^+^-C_13_H_10_O), 234 (22, M^+^-C_14_H_14_N_2_), 208 (4, M^+^-C_14_H_10_N_3_O^−^), 165 (1, M^+^-C_16_H_15_N_4_O), 146 (2, M^+^-C_19_H_12_N_3_O), 139 (7, M^+^-C_17_H_15_N_5_O), 88 (13, M^+^-C_22_H_20_N_4_O), 77 (21, M^+^-C_21_H_15_N_6_O) and 62 (2, M^+^-C_23_H_20_N_5_O); Anal. Calc. for C_27_H_20_N_6_O (444.49): C, 72.96; H, 4.54; N, 18.91%, found: C, 72.91; H, 4.51; N, 18.86%.

*(E)-3-{2-(2,5-Diphenylpyrazolo[1,5-c]pyrimidin-7-yl)hydrazono}-5-chloroindolin-2-one* (**3c**). Yield 86%; m.p. 320–322 °C; R_f_ 0.34 (3:1 benzene-EtOAc); IR (cm^−1^): 3459 (NH), 1710 (indole ring C=O), 1631 (pyrazole ring C=N), 1539 (pyrimidine ring C=N) and 1459 (pyrimidine ring C=C); ^1^H-NMR (DMSO-*d_6_*, δ_H_, ppm): 7.02 (d, 1H, aromatic-H), 7.16 (s, 1H, aromatic-H), 7.21 (s, 1H, pyrazole-H), 7.41–7.57 (m, 6H, aromatic-H), 7.68 (d, 1H, aromatic-H), 7.92 (s, 1H, pyrimidine-H), 8.11 (d, 2H, aromatic-H), 8.23 (d, 2H, aromatic-H), 11.36, 12.17 (s, 1H, exchangeable NH, OH) and 10.43, 14.19 (s, 1H, exchangeable NH); MS, *m/z* (%): 464 (29, M^+^), 436 (41, M^+^-N_2_), 359 (100, M^+^-C_7_H_7_N), 282 (2, M^+^-C_13_H_10_O), 234 (51, M^+^-C_13_H_11_ClN_2_), 228 (2, M^+^-C_14_H_10_N_3_O^−^), 166 (7, M^+^-C_19_H_12_N_3_O), 165 (5, M^+^-C_15_H_12_ClN_4_O), 139 (18, M^+^-C_16_H_12_ClN_5_O), 88 (19, M^+^-C_21_H_17_ClN_4_O), 77 (50, M^+^-C_20_H_12_ClN_6_O) and 62 (17, M^+^-C_22_H_17_ClN_5_O); Anal. Calc. for C_26_H_17_ClN_6_O (464.91): C, 67.17; H, 3.69; Cl, 7.63; N, 18.08%, found: C, 67.12; H, 3.65; Cl, 7.60; N, 17.95%.

#### 3.2.2. (Z)-3-{2-(2,5-Diphenylpyrazolo[1,5-c]pyrimidin-7-yl)hydrazono}indolin-2-ones **3a–c**

Method A: A suspension of (*E*)-3-{2-(2,5-diphenylpyrazolo[1,5-*c*]pyrimidin-7-yl)hydrazono}indolin-2-ones (*E*)-**3a–c** (0.0046 mol) in dioxane, xylene, pyridine, acetic acid or acetic anhydride (50 mL) was heated under reflux for twenty four hours. The products that separated out were filtered off, washed with ethanol, dried and crystallized from dioxane.

Method B: Stirring of (*E*)-3-{2-(2,5-diphenylpyrazolo[1,5-*c*]pyrimidin-7-yl)hydrazono}indolin-2-ones (*E*)-**3a–c** in conc. sulfuric acid (5.0 mL) was set below 15 °C and left for 2 hours. The reaction mixture was poured onto crushed ice and the separated product were filtered off, washed with water, dried and crystallized from dioxane.

The products from method A and method B showed completely similar TLC, mp, mixed mp, IR, ^1^H-NMR and MS spectra.

*(Z)-3-{2-(2,5-Diphenylpyrazolo[1,5-c]pyrimidin-7-yl)hydrazono}indolin-2-one* (**3a**). Yield 80%; m.p. 318–320 °C (crystallization from dioxane); R_f_ 0.66 (3:1 benzene-EtOAc); IR (cm^−1^): 3467 (NH), 1692 (indole ring C=O), 1626 (pyrazole ring C=N), 1557 (pyrimidine ring C=N) and 1458 (pyrimidine ring C=C); ^1^H-NMR (DMSO-*d_6_*, δ_H_, ppm): 7.01 (d, 1H, aromatic-H), 7.12 (t, 1H, aromatic-H), 7.19 (s, 1H, pyrazole-H), 7.37–7.71 (m, 8H, aromatic-H), 7.91 (s, 1H, pyrimidine-H), 8.11 (d, 2H, aromatic-H), 8.22 (d, 2H, aromatic-H), 11.27 (s, 1H, exchangeable NH) and 14.21 (s, 1H, exchangeable NH); MS, *m/z* (%): 430 (7, M^+^ ), 402 (1, M^+^-N_2_), 325 (14, M^+^-C_7_H_7_N), 248 (1, M^+^-C_13_H_10_O), 234 (2, M^+^-C_13_H_12_N_2_), 194 (1, M^+^-C_14_H_10_N_3_O^−^), 165 (2, M^+^-C_15_H_13_N_4_O), 139 (10, M^+^-C_16_H_13_N_5_O), 132 (1, M^+^-C_19_H_12_N_3_O), 88 (48, M^+^-C_21_H_18_N_4_O), 77 (100, M^+^-C_20_H_13_N_6_O) and 62 (17, M^+^-C_22_H_18_N_5_O); Anal. Calc. for C_26_H_18_N_6_O (430.46): C, 72.55; H, 4.21; N, 19.52%, found: C, 72.56; H, 4.12; N, 19.12%.

*(Z)-3-{2-(2,5-Diphenylpyrazolo[1,5-c]pyrimidin-7-yl)hydrazono}-5-methylindolin-2-one* (**3b**). Yield 86%; m.p. 328–330 °C (crystallization from dioxane); R_f_ 0.69 (3:1 benzene-EtOAc); IR (cm^−1^): 3451 (NH), 1684 (indole ring C=O), 1629 (pyrazole ring C=N), 1558 (pyrimidine ring C=N) and 1459 (pyrimidine ring C=C); ^1^H-NMR (DMSO-*d_6_*, δ_H_, ppm): 2.33 (s, 3H, CH_3_), 6.87 (d, 1H aromatic-H) 7.17 (s, 1H, aromatic-H), 7.18 (s, 1H, pyrazole-H), 7.43–7.53 (m, 7H, aromatic-H), 7.88 (s, 1H, pyrimidine-H), 8.11 (d, 2H, aromatic-H), 8.22 (d, 2H, aromatic-H), 11.14 (s, 1H, exchangeable NH) and 14.23 (s, 1H, exchangeable NH); MS, *m/z* (%): 444 (30, M^+^), 416 (40, M^+^-N_2_), 339 (100, M^+^-C_7_H_7_N), 262 (2, M^+^-C_13_H_10_O), 234 (31, M^+^-C_14_H_14_N_2_), 208(5, M^+^-C_14_H_10_N_3_O^−^), 165 (3, M^+^-C_16_H_15_N_4_O), 146 (8, M^+^-C_19_H_12_N_3_O), 139 (4, M^+^-C_17_H_15_N_5_O), 88 (22, M^+^-C_22_H_20_N_4_O), 77 (40, M^+^-C_21_H_15_N_6_O) and 62 (10, M^+^-C_23_H_20_N_5_O); Anal. Calc. for C_27_H_20_N_6_O (444.49): C, 72.96; H, 4.54; N, 18.91%, found: C, 72.89; H, 4.52; N, 18.82%. 

*(Z)-3-{2-(2,5-Diphenylpyrazolo[1,5-c]pyrimidin-7-yl)hydrazono}-5-chloroindolin-2-one* (**3c**). Yield 91%; m.p. 312–314 °C (crystallization from dioxane); R_f_ 0.63 (3:1 benzene-EtOAc); IR (cm^−1^): 3464 (NH), 1689 (indole ring C=O), 1628 (pyrazole ring C=N), 1556 (pyrimidine ring C=N) and 1455 (pyrimidine ring C=C); ^1^H-NMR (DMSO-*d_6_*, δ_H_, ppm): 7.03 (d, 1H, aromatic-H), 7.15 (s, 1H, aromatic-H), 7.22 (s, 1H, pyrazole-H), 7.43–7.59 (m, 6H, aromatic-H), 7.71 (d, 1H, aromatic-H), 7.95 (s, 1H, pyrimidine-H), 8.13 (d, 2H, aromatic-H), 8.27 (d, 2H, aromatic-H), 11.37 (s, 1H, exchangeable NH) and 14.22 (s, 1H, exchangeable NH); MS, *m/z* (%): 464 (26, M^+^), 436 (41, M^+^-N_2_), 359 (100, M^+^-C_7_H_7_N), 282 (3, M^+^-C_13_H_10_O), 234 (56, M^+^-C_13_H_11_ClN_2_), 228 (2, M^+^-C_14_H_10_N_3_O^−^), 166 (9, M^+^-C_19_H_12_N_3_O), 165 (6, M^+^-C_15_H_12_ClN_4_O), 139 (21, M^+^-C_16_H_12_ClN_5_O), 88 (39, M^+^-C_21_H_17_ClN_4_O), 77 (72, M^+^-C_20_H_12_ClN_6_O) and 62 (23, M^+^-C_22_H_17_ClN_5_O); Anal. Calc. for C_26_H_17_ClN_6_O (464.91): C, 67.17; H, 3.69; Cl, 7.63; N, 18.08%, found: C, 67.11; H, 3.63; Cl, 7.58; N, 17.91%.

#### 3.2.3. (Z)-3-{2-(3-Bromo-2,5-diphenylpyrazolo[1,5-c]pyrimidin-7-yl)hydrazono}indolin-2-ones **4a–c**

A solution of bromine (0.06 mL, 0.0012 mol) in acetic acid (10 mL) was gradually added to a suspension of (*E *or*Z*)-3-{2-(2,5-diphenylpyrazolo[1,5-*c*]pyrimidin-7-yl)hydrazono}indolin-2-ones **3a‑c** (0.0010 mol) in acetic acid (10 mL) with stirring for three hours at room temperature. The reaction mixture was then poured onto crushed ice, filtered off, washed with water, dried and crystallized from dioxane as orange needles.

*(Z)-3-{2-(3-Bromo-2,5-diphenylpyrazolo[1,5-c]pyrimidin-7-yl)hydrazono}indolin-2-one* (**4a**) Yield 96%; m.p. 288–290 °C; R_f_ 0.65 (3:1 benzene-EtOAc); IR (cm^−1^): 3460 (NH), 1687 (indole ring C=O), 1622 (pyrazole ring C=N), 1559 (pyrimidine ring C=N) and 1456 (pyrimidine ring C=C); ^1^H-NMR (DMSO-*d_6_*, δ_H_, ppm): 6.98 (d, 1H, aromatic-H), 7.13 (t, 1H, aromatic-H), 7.39–7.71 (m, 8H, aromatic-H), 7.75 (s, 1H, pyrimidine-H), 8.05 (d, 2H, aromatic-H), 8.30 (d, 2H, aromatic-H), 11.29 (s, 1H, exchangeable NH) and 14.17 (s, 1H, exchangeable NH); MS, *m/z* (%): 510 (64, M^+^), 481 (26, M^+^-HN_2_), 405 (52, M^+^-C_7_H_7_N), 403 (91, M^+^-C_6_H_7_N_2_), 325 (100, M^+^-C_7_H_7_BrN), 312 10, M^+^-C_12_H_12_N_3_), 271 (27, M^+^-C_14_H_13_N_3_O^−^), 243 (23, M^+^-C_14_H_13_N_5_O), 234 (27, M^+^-C_13_H_12_BrN_2_), 165 (4, M^+^-C_15_H_13_BrN_4_O), 140 (4, M^+^-C_21_H_18_N_6_O), 139 (23, M^+^-C_16_H_13_BrN_5_O), 131 (9, M^+^-C_19_H_13_BrN_3_O), 88 (38, M^+^-C_21_H_18_BrN_4_O), 76 (90, M^+^-C_20_H_14_BrN_6_O) and 62 (35, M^+^-C_22_H_18_BrN_5_O); Anal. Calc. for C_26_H_17_BrN_6_O (509.36): C, 61.31; H, 3.36; Br, 15.69; N, 16.50%, found: C, 61.27; H, 3.32; Br, 15.52; N, 16.33%.

*(Z)-3-{2-(3-Bromo-2,5-diphenylpyrazolo[1,5-c]pyrimidin-7-yl)hydrazono}-5-methylindolin-2-one* (**4b**) Yield 97%; m.p. 320–322 °C; R_f_ 0.77 (3:1 benzene-EtOAc); IR (cm^−1^): 3465 (NH), 1689 (indole ring C=O), 1628 (pyrazole ring C=N), 1559 (pyrimidine ring C=N) and 1451 (pyrimidine ring C=C); ^1^H-NMR (DMSO-*d_6_*, δ, ppm): 2.36 (s, 3H, CH_3_), 6.88 (d, 1H aromatic-H) 7.19 (s, 1H, aromatic-H), 7.52–7.62 (m, 7H, aromatic-H), 7.73 (s, 1H, pyrimidine-H), 8.09 (d, 2H, aromatic-H), 8.16 (d, 2H, aromatic-H), 11.17 (s, 1H, exchangeable NH) and 14.22 (s, 1H, exchangeable NH); MS, *m/z* (%): 524 (98, M^+^), 495 (50, M^+^-HN_2_), 419 (77, M^+^-C_7_H_7_N), 417 (100, M^+^-C_6_H_7_N_2_), 339 (28, M^+^-C_7_H_7_BrN), 312 (22, M^+^-C_13_H_14_N_3_), 285 (19, M^+^-C_14_H_13_N_3_O^−^), 243 (11, M^+^-C_15_H_15_N_5_O), 234 (16, M^+^-C_14_H_14_BrN_2_), 165 (6, M^+^-C_16_H_15_BrN_4_O), 145 (14, M^+^-C_19_H_13_BrN_3_O), 140 (6, M^+^-C_22_H_20_N_6_O), 139 (28, M^+^-C_17_H_15_BrN_5_O), 88 (45, M^+^-C_22_H_20_BrN_4_O), 76 (95, M^+^-C_21_H_16_BrN_6_O) and 62 (29, M^+^-C_23_H_20_BrN_5_O); Anal. Calc. for C_27_H_19_BrN_6_O (523.38): C, 61.96; H, 3.66; Br, 15.27; N, 16.06%, found: C, 61.89; H, 3.63; Br, 15.18; N, 15.83%.

*(Z)-3-{2-(3-Bromo-2,5-diphenylpyrazolo[1,5-c]pyrimidin-7-yl)hydrazono}-5-chloroindolin-2-one* (**4c**) Yield 94%; m.p. 304–306 °C; R_f_ 0.75 (3:1 benzene-EtOAc); IR (cm^−1^): 3451 (NH), 1684 (indole ring C=O), 1630 (pyrazole ring C=N), 1560 (pyrimidine ring C=N) and 1447 (pyrimidine ring C=C); ^1^H-NMR (DMSO-*d_6_*, δ, ppm): 7.03 (d, 1H, aromatic-H), 7.19 (s, 1H, aromatic-H), 7.42–7.57 (m, 6H, aromatic-H), 7.69 (d, 1H, aromatic-H), 7.93 (s, 1H, pyrimidine-H), 8.11 (d, 2H, aromatic-H), 8.25 (d, 2H, aromatic-H), 11.36 (s, 1H, exchangeable NH) and 14.20 (s, 1H, exchangeable NH); MS, *m/z* (%): 544 (53, M^+^), 515 (27, M^+^-HN_2_), 439 (10, M^+^-C_7_H_7_N), 437 (100, M^+^-C_6_H_7_N_2_), 359 (13, M^+^-C_7_H_7_BrN), 312 (11, M^+^-C_12_H_11_ClN_3_), 305 (1, M^+^-C_14_H_13_N_3_O^−^), 243 (18, M^+^-C_14_H_12_ClN_5_O), 234 (2, M^+^-C_13_H_11_BrClN_2_), 165 (15, M^+^-C_15_H_12_BrClN_4_O), 140 (3, M^+^-C_21_H_17_ClN_6_O), 139 (20, M^+^-C_16_H_12_BrClN_5_O), 88 (14, M^+^-C_21_H_17_BrClN_4_O), 76 (53, M^+^-C_20_H_13_BrClN_6_O) and 62 (13, M^+^-C_22_H_17_BrClN_5_O); Anal. Calc. for C_26_H_16_BrClN_6_O (543.80): C, 57.42; H, 2.97; Br, 14.69; Cl, 6.52; N, 15.45%, found: C, 57.38; H, 2.95; Br, 14.51; Cl, 6.31; N, 15.23%.

#### 3.2.4. (E)-3-{2-(3-Bromo-2,5-diphenylpyrazolo[1,5-c]pyrimidin-7-yl)hydrazono}indolin-2-ones **9a–c**

A solution of 3-bromo-2,5-diphenyl-7-hydrazinopyrazolo[1,5-*c*]pyrimidine [45] (**10**, 0.46 g, 0.0012 mol) in dioxane (10 mL) was stirred with isatin (or isatin derivatives) **2** (0.0015 mol) for 24 h at room temperature. The products that separated out were filtered off, washed with methanol and dried.

*(E)-3-{2-(3-Bromo-2,5-diphenylpyrazolo[1,5-c]pyrimidin-7-yl)hydrazono}indolin-2-one* (**9a**). Yield 81%; m.p. 312–314 °C; R_f_ 0.27 (3:1 benzene-EtOAc); IR (cm^−1^): 3442 (NH), 1707 (indole ring C=O), 1626 (pyrazole ring C=N), 1555 (pyrimidine ring C=N) and 1453 (pyrimidine ring C=C); ^1^H-NMR (DMSO-*d_6_*, δ, ppm): 7.87–8.05 (m, 10H, aromatic-H), 7.74 (s, 1H, pyrimidine-H), 8.08 (d, 2H, aromatic-H), 8.25 (d, 2H, aromatic-H), 11.21, 11.29 (s, 1H, exchangeable NH, OH) and 10.92, 14.16 (s, 1H, exchangeable NH); MS, *m/z* (%): 510 (35, M^+^), 481 (28, M^+^-HN_2_), 405 (81, M^+^-C_7_H_7_N), 403 (100, M^+^-C_6_H_7_N_2_), 325 (33, M^+^-C_7_H_7_BrN), 312 (15, M^+^-C_12_H_12_N_3_), 271 (3, M^+^-C_14_H_13_N_3_O^−^), 243 (13, M^+^-C_14_H_13_N_5_O), 234 (16, M^+^-C_13_H_12_BrN_2_), 165 (7, M^+^-C_15_H_13_BrN_4_O), 140 (4, M^+^-C_21_H_18_N_6_O), 139 (22, M^+^-C_16_H_13_BrN_5_O), 131 (7, M^+^-C_19_H_13_BrN_3_O), 88 (47, M^+^-C_21_H_18_BrN_4_O), 76 (73, M^+^-C_20_H_14_BrN_6_O) and 62 (18, M^+^-C_22_H_18_BrN_5_O); Anal. Calc. for C_26_H_17_BrN_6_O (509.36): C, 61.31; H, 3.36; Br, 15.69; N, 16.50%, found: C, 61.27; H, 3.30; Br, 15.46; N, 16.38%.

*(E)-3-{2-(3-Bromo-2,5-diphenylpyrazolo[1,5-c]pyrimidin-7-yl)hydrazono}-5-methylindolin-2-one* (**9b**). Yield 79%; m.p. 300–302 °C; R_f_ 0.43 (3:1 benzene-EtOAc); IR (cm^−1^): 3451 (NH), 1698 (indole ring C=O), 1630 (pyrazole ring C=N), 1543 (pyrimidine ring C=N) and 1460 (pyrimidine ring C=C); MS, *m/z* (%): 524 (81, M^+^), 495 (40, M^+^-HN_2_), 419 (69, M^+^-C_7_H_7_N), 417 (86, M^+^-C_6_H_7_N_2_), 339 (32, M^+^-C_7_H_7_BrN), 312 (17, M^+^-C_13_H_14_N_3_), 285 (26, M^+^-C_14_H_13_N_3_O^−^), 243 (15, M^+^-C_15_H_15_N_5_O), 234 (16, M^+^-C_14_H_14_BrN_2_), 165 (7, M^+^-C_16_H_15_BrN_4_O), 145 (11, M^+^-C_19_H_13_BrN_3_O), 140 (12, M^+^-C_22_H_20_N_6_O), 139 (30, M^+^-C_17_H_15_BrN_5_O), 88 (38, M^+^-C_22_H_20_BrN_4_O), 76 (100, M^+^-C_21_H_16_BrN_6_O) and 62 (21, M^+^-C_23_H_20_BrN_5_O); Anal. Calc. for C_27_H_19_BrN_6_O (523.38): C, 61.96; H, 3.66; Br, 15.27; N, 16.06%, found: C, 61.81; H, 3.51; Br, 15.05; N, 15.76%.

*(E)-3-{2-(3-Bromo-2,5-diphenylpyrazolo[1,5-c]pyrimidin-7-yl)hydrazono}-5-chloroindolin-2-one *(**9c**). Yield 79%; m.p. 308–310 °C; R_f_ 0.42 (3:1 benzene-EtOAc); IR (cm^−1^): 3464 (NH), 1700 (indole ring C=O), 1629 (pyrazole ring C=N), 1533 (pyrimidine ring C=N) and 1449 (pyrimidine ring C=C); MS, *m/z* (%): 544 (100, M^+^), 515 (44, M^+^-HN_2_), 439 (91, M^+^-C_7_H_7_N), 437 (94, M^+^-C_6_H_7_N_2_), 359 (29, M^+^-C_7_H_7_BrN), 312 (18, M^+^-C_12_H_11_ClN_3_), 305 (1, M^+^-C_14_H_13_N_3_O^−^), 243 (18, M^+^-C_14_H_12_ClN_5_O), 234 (9, M^+^-C_13_H_11_BrClN_2_), 165 (12, M^+^-C_15_H_12_BrClN_4_O), 140 (5, M^+^-C_21_H_17_ClN_6_O), 139 (36 M^+^-C_16_H_12_BrClN_5_O), 88 (24, M^+^-C_21_H_17_BrClN_4_O), 76 (98, M^+^-C_20_H_13_BrClN_6_O) and 62 (35, M^+^-C_22_H_17_BrClN_5_O); Anal. Calc. for C_26_H_16_BrClN_6_O (543.80): C, 57.42; H, 2.97; Br, 14.69; Cl, 6.52; N, 15.45%, found: C, 57.21; H, 2.82; Br, 14.48; Cl, 6.25; N, 15.27%.

#### 3.2.5. (Z)-3-{2-(3-Iodo-2,5-diphenylpyrazolo[1,5-c]pyrimidin-7-yl)hydrazono}indolin-2-ones **5a–c**

A solution of iodine monochloride (0.20 g, 0.0012 mol) in acetic acid (10 mL) was gradually added to a suspension of (*E* or *Z*)-3**a–c** (0.001 mol) in acetic acid (10 mL) with stirring for three hours at room temperature. The reaction mixture was then poured onto crushed ice and the products that separated out were filtered off, washed with water, dried and crystallized from dioxan as orange needles.

*(Z)-3-{2-(3-Iodo-2,5-diphenylpyrazolo[1,5-c]pyrimidin-7-yl)hydrazono}indolin-2-one* (**5a**). Yield 92%; m.p. 280–282 °C; IR (cm^−1^): 3454 (NH), 1684 (indole ring C=O), 1622 (pyrazole ring C=N), 1561 (pyrimidine ring C=N) and 1455 (pyrimidine ring C=C); ^1^H-NMR (DMSO-*d_6_*, δ, ppm): 6.96 (d, 1H aromatic-H) 7.12 (t, 1H, aromatic-H), 7.36–7.69 (m, 8H, aromatic-H), 7.61 (s, 1H, pyrimidine-H), 8.00 (d, 2H, aromatic-H), 8.27 (d, 2H, aromatic-H), 11.27 (s, 1H, exchangeable NH) and 14.12 (s, 1H, exchangeable NH); MS, *m/z* (%): 557 (95, M^+^), 529 (24, M^+^-N_2_), 452 (100, M^+^-C_7_H_7_N), 374 (1, M^+^-C_13_H_11_O), 360 (22, M^+^-C_12_H_11_N_3_), 325 (41, M^+^-C_7_H_7_IN), 320 (1, M^+^-C_14_H_11_N_3_O), 257 (5, M^+^-C_19_H_16_N_4_^−^), 234 (8, M^+^-C_13_H_12_IN_2_), 188 (10, M^+^-C_21_H_17_N_6_O), 165 (7, M^+^-C_15_H_13_IN_4_O), 139 (41, M^+^-C_16_H_13_IN_5_O), 131 (8, M^+^-C_19_H_13_IN_3_O), 88 (7, M^+^-C_21_H_18_IN_4_O), 76 (96, M^+^-C_20_H_14_IN_6_O) and 62 (35, M^+^-C_22_H_18_IN_5_O); Anal. Calc. for C_26_H_17_IN_6_O (556.36): C, 56.13; H, 3.08; I, 22.81; N, 15.11%, found: C, 55.89; H, 2.99; I, 22.45; N, 14.78%.

*(Z)-3-{2-(3-Iodo-2,5-diphenylpyrazolo[1,5-c]pyrimidin-7-yl)hydrazono}-5-methylindolin-2-one* (**5b**). Yield 98%; m.p. 314–316 °C; IR (cm^−1^): 3443 (NH), 1684 (indole ring C=O), 1630 (pyrazole ring C=N), 1564 (pyrimidine ring C=N) and 1460 (pyrimidine ring C=C); MS, *m/z* (%): 571 (67, M^+^ ), 543 (14, M^+^-N_2_), 466 (44, M^+^-C_7_H_7_N), 388 (2, M^+^-C_13_H_11_O), 360 (3, M^+^-C_13_H_13_N_3_), 339 (25, M^+^-C_7_H_7_IN), 334 (8, M^+^-C_14_H_11_N_3_O), 257 (4, M^+^-C_20_H_18_N_4_^−^), 234 (3, M^+^-C_14_H_14_IN_2_), 188 (5, M^+^-C_22_H_19_N_6_O), 165 (5, M^+^-C_16_H_15_IN_4_O), 145 (7, M^+^-C_19_H_13_IN_3_O), 139 (11, M^+^-C_17_H_15_IN_5_O), 88 (11, M^+^-C_22_H_20_IN_4_O), 76 (100, M^+^-C_21_H_16_IN_6_O) and 62 (13, M^+^-C_23_H_20_IN_5_O); Anal. Calc. for C_27_H_19_IN_6_O (570.38): C, 56.85; H, 3.36; I, 22.25; N, 14.73%, found: C, 56.73; H, 3.24; I, 21.83; N, 14.47%.

*(Z)-3-{2-(3-Iodo-2,5-diphenylpyrazolo[1,5-c]pyrimidin-7-yl)hydrazono}-5-chloroindolin-2-one* (**5c**). Yield 97%; m.p. 306–308 °C; IR (cm^−1^): 3466 (NH), 1677 (indole ring C=O), 1625 (pyrazole ring C=N), 1558 (pyrimidine ring C=N) and 1447 (pyrimidine ring C=C); ^1^H-NMR (DMSO-*d_6_*, δ, ppm): 7.01 (d, 1H, aromatic-H), 7.16 (s, 1H, aromatic-H), 7.39–7.66 (m, 7H, aromatic-H), 7.89 (s, 1H, pyrimidine-H), 8.11 (d, 2H, aromatic-H), 8.24 (d, 2H, aromatic-H), 11.33 (s, 1H, exchangeable NH) and 14.17 (s, 1H, exchangeable NH); MS, *m/z* (%): 591 (33, M^+^), 563 (12, M^+^-N_2_), 486 (44, M^+^-C_7_H_7_N), 408 (1, M^+^-C_13_H_11_O), 360 (19, M^+^-C_12_H_10_ClN_3_), 359 (63, M^+^-C_7_H_7_IN), 354 (10, M^+^-C_14_H_11_N_3_O), 257 (9, M^+^-C_19_H_15_ClN_4_^−^), 234 (20, M^+^-C_13_H_11_ClIN_2_), 188 (10, M^+^-C_21_H_16_ClN_6_O), 165 (12, M^+^-C_15_H_12_ClIN_4_O), 139 (43, M^+^-C_16_H_12_ClIN_5_O), 88 (32, M^+^-C_21_H_17_ClIN_4_O), 76 (100, M^+^-C_20_H_13_ClIN_6_O) and 62 (27, M^+^-C_22_H_17_ClIN_5_O); Anal. Calc. for C_26_H_16_ClIN_6_O (590.80): C, 52.86; H, 2.73; Cl, 6.00; I, 21.48; N, 14.22%, found: C, 52.77; H, 2.62; Cl, 4.68; I, 21.20; N, 13.93%.

#### 3.2.6. (Z)-3-{2-(3-Nitro-2,5-diphenylpyrazolo[1,5-c]pyrimidin-7-yl)hydrazono}indolin-2-ones **6a–c**

A mixture of nitric acid (d 1.14, 1 mL) and sulfuric acid (d 1.84, 1 mL) in glacial acetic acid (10 mL) was added gradually to a suspension of (*E* or *Z*)-**3a–c** (0.001 mol) in acetic acid (10 mL) with stirring for three hours at room temperature. The reaction mixture was then poured onto crushed ice and the products that separated out were filtered off, washed with water, dried and crystallized from dioxane as orange needles.

*(Z)-3-{2-(3-Nitro-2,5-diphenylpyrazolo[1,5-c]pyrimidin-7-yl)hydrazono}indolin-2-one* (**6a**). Yield 91%; m.p. 268–270 °C; IR (cm^−1^): 3460 (NH), 1708 (indole ring C=O), 1624 (pyrazole ring C=N), 1560 (pyrimidine ring C=N), 1473 (pyrimidine ring C=C), and 1416, 1341 (NO_2_); MS, *m/z* (%): 476 (1, M^+^), 447 (1, M^+^-HN_2_), 371 (5, M^+^-C_7_H_7_N), 370 (2, M^+^-C_6_H_6_N_2_), 325 (5, M^+^-C_7_H_7_N_2_O_2_), 291 (3, M^+^-C_13_H_13_O), 279 (3, M^+^-C_12_H_11_N_3_^−^), 237 (3, M^+^-C_14_H_13_N_3_O), 234 (5, M^+^-C_13_H_12_N_3_O_2_), 210 (3, M^+^-C_14_H_12_N_5_O), 165 (5, M^+^-C_15_H_13_N_5_O_3_), 139 (3, M^+^-C_16_H_13_N_6_O_3_), 131 (5, M^+^-C_19_H_13_N_4_O_3_), 107 (9, M^+^-C_21_H_17_N_6_O), 88 (8, M^+^-C_21_H_18_N_5_O_3_), 76 (7, M^+^-C_20_H_14_N_7_O_3_) and 62 (4, M^+^-C_22_H_18_N_6_O_3_); Anal. Calc. for C_26_H_17_N_7_O_3_ (475.46): C, 65.68; H, 3.60; N, 20.62%, found: C, 65.56; H, 3.57; N, 20.40%.

*(Z)-3-{2-(3-Nitro-2,5-diphenylpyrazolo[1,5-c]pyrimidin-7-yl)hydrazono}-5-methylindolin-2-one* (**6b**). Yield 95%; m.p. 318–320 °C; IR (cm^−1^): 3465 (NH), 1697 (indole ring C=O), 1635 (pyrazole ring C=N), 1564 (pyrimidine ring C=N), 1487 (pyrimidine ring C=C), and 1419, 1381 (NO_2_); MS, *m/z* (%): 490 (20, M^+^), 461 (7, M^+^-HN_2_), 385 (35, M^+^-C_7_H_7_N), 384 (3, M^+^-C_6_H_6_N_2_), 339 (5, M^+^-C_7_H_7_N_2_O_2_), 305 (1, M^+^-C_13_H_13_O), 279 (1, M^+^-C_13_H_13_N_3_^−^), 251 (4, M^+^-C_14_H_13_N_3_O), 234 (8, M^+^-C_14_H_14_N_3_O_2_), 210 (2, M^+^-C_15_H_14_N_5_O), 165 (5, M^+^-C_16_H_15_N_5_O_3_), 145 (28, M^+^-C_19_H_13_N_4_O_3_), 139 (8, M^+^-C_17_H_15_N_6_O_3_), 107 (24, M^+^-C_22_H_19_N_6_O), 88 (11, M^+^-C_22_H_20_N_5_O_3_), 76 (85, M^+^-C_21_H_16_N_7_O_3_) and 62 (16, M^+^-C_23_H_20_N_6_O_3_); Anal. Calc. for C_27_H_19_N_7_O_3_ (489.48): C, 66.25; H, 3.91; N, 20.03%, found: C, 66.02; H, 3.72; N, 19.84%.

*(Z)-3-{2-(3-Nitro-2,5-diphenylpyrazolo[1,5-c]pyrimidin-7-yl)hydrazono}-5-chloroindolin-2-one* (**6c**). Yield 97%; m.p. 330–332 °C; IR (cm^−1^): 3460 (NH), 1693 (indole ring C=O), 1629 (pyrazole ring C=N), 1559 (pyrimidine ring C=N), 1487 (pyrimidine ring C=C), and 1416, 1363 (NO_2_); MS, *m/z* (%): 510 (1, M^+^), 481 (3, M^+^-HN_2_), 405 (1, M^+^-C_7_H_7_N), 404 (1, M^+^-C_6_H_6_N_2_), 359 (1, M^+^-C_7_H_7_N_2_O_2_), 325 (5, M^+^-C_13_H_13_O), 279 (1, M^+^-C_12_H_10_ClN_3_^−^), 271 (1, M^+^-C_14_H_13_N_3_O), 234 (1, M^+^-C_13_H_11_ClN_3_O_2_), 210 (1, M^+^-C_14_H_11_ClN_5_O), 165 (2, M^+^-C_15_H_12_ClN_5_O_3_), 139 (3, M^+^-C_16_H_12_ClN_6_O_3_), 107 (1, M^+^-C_21_H_16_ClN_6_O), 88 (3, M^+^-C_21_H_17_ClN_5_O_3_), 76 (100, M^+^-C_20_H_13_ClN_7_O_3_) and 62 (13, M^+^-C_22_H_17_ClN_6_O_3_); Anal. Calc. for C_26_H_16_ClN_7_O_3_ (509.90): C, 61.24; H, 3.16; Cl, 6.95; N, 19.23%, found: C, 61.19; H, 3.02; Cl, 6.62; N, 18.91%.

#### 3.2.7. (Z)-3-{2-(3-Phenyldiazenyl-2,5-diphenylpyrazolo[1,5-c]pyrimidin-7-yl)hydrazono}indolin-2-ones **7a–c**

An aqueous sodium hydroxide solution (10%, 8 mL) was added to a suspension of (*E* or *Z*)-**3a–c** (0.001 mol) in ethanol (15 mL). The reaction mixture was cooled to 5 °C and gradually treated with a solution of benzendiazonium chloride (prepared from 1 mL of aniline) with stirring for one hour. The target products that separated out were collected by filtration and crystallized from dioxan as reddish-brown needles.

*(Z)-3-{2-(3-Phenyldiazenyl-2,5-diphenylpyrazolo[1,5-c]pyrimidin-7-yl)hydrazono}indolin-2-one* (**7a**). Yield 94%; m.p. 298–300 °C; IR (cm^−1^): 3463 (NH), 1693 (indole ring C=O), 1625 (pyrazole ring C=N), 1557 (pyrimidine ring C=N) and 1458 (pyrimidine ring C=C); MS, *m/z* (%): 534(1, M^+^), 506 (1, M^+^-N_2_), 431 (29, M^+^-C_7_H_5_N), 430 (2, M^+^-C_6_H_4_N_2_), 353 (1, M^+^-C_13_H_9_O), 339 (1, M^+^-C_12_H_9_N_3_), 325 (69, M^+^-C_13_H_11_N_3_), 298 (1, M^+^-C_14_H_10_N_3_O), 270 (7, M^+^-C_14_H_10_N_5_O^−^), 234 (14, M^+^-C_19_H_16_N_4_), 167 (6, M^+^-C_21_H_15_N_6_O), 165 (5, M^+^-C_21_H_17_N_6_O), 139 (17, M^+^-C_22_H_17_N_7_O), 131 (6, M^+^-C_25_H_17_N_5_O), 88 (44, M^+^-C_27_H_22_N_6_O), 76 (100, M^+^-C_26_H_18_N_8_O) and 62 (21, M^+^-C_28_H_22_N_7_O); Anal. Calc. for C_32_H_22_N_8_O (534.57): C, 71.90; H, 4.15; N, 20.96%, found: C, 71.79; H, 3.97; N, 20.62%. 

*(Z)-3-{2-(3-Phenydiazenyl-2,5-diphenylpyrazolo[1,5-c]pyrimidin-7-yl)hydrazono}-5-methyl-indolin-2-one *(**7b**). Yield 94%; m.p. 338–340 °C; IR (cm^−1^): 3460 (NH), 1689 (indole ring C=O), 1629 (pyrazole ring C=N), 1557 (pyrimidine ring C=N) and 1455 (pyrimidine ring C=C); MS, *m/z* (%): 548 (1, M^+^), 520 (3, M^+^-N_2_), 445 (40, M^+^-C_7_H_5_N), 444 (3, M^+^-C_6_H_4_N_2_), 367 (1, M^+^-C_13_H_9_O), 339 (100, M^+^-C_13_H_11_N_3_), 312 (2, M^+^-C_14_H_10_N_3_O), 270 (11, M^+^-C_15_H_12_N_5_O^−^), 234 (29, M^+^-C_20_H_18_N_4_), 167 (4, M^+^-C_22_H_17_N_6_O), 165 (3, M^+^-C_22_H_19_N_6_O), 145 (6, M^+^-C_25_H_17_N_5_O), 139 (16, M^+^-C_23_H_19_N_7_O), 88 (52, M^+^-C_28_H_24_N_6_O), 76 (7, M^+^-C_27_H_20_N_8_O) and 62 (8, M^+^-C_29_H_24_N_7_O); Anal. Calc. for C_33_H_24_N_8_O (548.60): C, 72.25; H, 4.41; N, 20.43%, found: C, 72.12; H, 4.20; N, 20.22%.

*(Z)-3-{2-(3-Phenyldiazenyl-2,5-diphenylpyrazolo[1,5-c]pyrimidin-7-yl)hydrazono}-5-chloro-indolin-2-one *(**7c**). Yield 94%; m.p. 328–330 °C; IR (cm^−1^): 3460 (NH), 1690 (indole ring C=O), 1626 (pyrazole ring C=N), 1553 (pyrimidine ring C=N) and 1451 (pyrimidine ring C=C); MS, *m/z* (%): 568 (2, M^+^), 540 (3, M^+^-N_2_), 465 (45, M^+^-C_7_H_5_N), 464 (1, M^+^-C_6_H_4_N_2_), 387 (5, M^+^-C_13_H_9_O), 359 (100, M^+^-C_13_H_11_N_3_), 339 (5, M^+^-C_12_H_8_ClN_3_), 332 (4, M^+^-C_14_H_10_N_3_O), 270 (17, M^+^-C_14_H_9_ClN_5_O^−^), 234 (46, M^+^-C_19_H_15_ClN_4_), 167 (8, M^+^-C_21_H_14_ClN_6_O), 165 (4, M^+^-C_21_H_16_ClN_6_O), 139 (18, M^+^-C_22_H_16_ClN_7_O), 88 (12, M^+^-C_27_H_21_ClN_6_O), 76 (50, M^+^-C_26_H_17_ClN_8_O) and 62 (13, M^+^-C_28_H_21_ClN_7_O); Anal. Calc. for C_32_H_21_ClN_8_O (569.02): C, 67.55; H, 3.72; Cl, 6.23; N, 19.69%, found: C, 67.36; H, 3.61; Cl, 5.82; N, 19.42%.

#### 3.2.8. 2,5-Diphenylindolo[2,3-e]pyrazolo[1',5':3",4"]pyrimido[2",1"-c][1,2,4]triazines **13a–c**

A solution of (*E*)-**3a–c** (0.0023 mol) in phosphorus oxychloride (15 mL) was heated at 70–80 °C for two hours. The mixture was cooled, poured onto crushed ice and made alkaline (pH = 9) with potassium hydrogen carbonate. The target productswere filtered off, washed with water, dried and crystallized from dimethylformamide as reddish-brown needles.

*2,5-Diphenylindolo[2,3-e]pyrazolo[1',5':3",4"]pyrimido[2",1"-c][1,2,4]triazines* (**13a**). Yield 93%; m.p. 308–310 °C; IR (cm^−1^): 1647 (indole ring C=N), 1624 (pyrazole ring C=N), 1535 (triazine ring C=N) and 1470 (pyrimidine ring C=C); ^1^H-NMR (DMSO-*d_6_*, δ, ppm): 7.24 (d, 1H, aromatic-H), 7.53 (s, 1H, pyrazole-H), 7.33–7.55 (m, 9H, aromatic-H), 7.91 (s, 1H, pyrimidine-H), 8.13 (d, 2H, aromatic-H), 8.20 (d, 2H, aromatic-H); MS, *m/z* (%): 412 (64, M^+^), 335 (2, M^+^-C_6_H_5_), 307 (15, M^+^-C_6_H_5_N_2_), 281 (13, M^+^-C_8_H_7_N_2_), 253 (8, M^+^-C_8_H_7_N_4_), 228 (7, M^+^-C_12_H_12_N_2_^−^), 217 (8, M^+^-C_13_H_11_N_2_), 191 (4, M^+^-C_14_H_11_N_3_), 176 (7, M^+^-C_14_H_12_N_4_), 150 (12, M^+^-C_15_H_12_N_5_), 114 (17, M^+^-C_20_H_16_N_3_), 88 (27, M^+^-C_21_H_16_N_4_), 76 (100, M^+^-C_20_H_12_N_6_), 62 (11, M^+^-C_22_H_16_N_5_) and 50 (34, M^+^-C_23_H_16_N_5_); Anal. Calc. for C_26_H_16_N_6_ (412.45): C, 75.71; H, 3.91; N, 20.38%, found: C, 75.49; H, 3.76; N, 20.08%.

*2,5-Diphenyl-10-methylindolo[2,3-e]pyrazolo[1',5':3",4"]pyrimido[2",1"-c][1,2,4]triazines* (**13b**). Yield 96%; m.p. 286–288 °C; IR (cm^−1^): 1648 (indole ring C=N), 1604 (pyrazole ring C=N), 1542 (triazine ring C=N) and 1474 (pyrimidine ring C=C); ^1^H-NMR (DMSO-*d*_6_, δ, ppm): 2.44 (s, 3H, CH_3_), 7.11 (d, 1H, aromatic-H), 7.42 (s, 1H, pyrazole-H), 7.33–7.53 (m, 8H, aromatic-H), 7.55 (s, 1H, pyrimidine-H), 8.01 (d, 2H, aromatic-H), 8.13 (d, 2H, aromatic-H); MS, *m/z* (%): 426 (100, M^+^), 349 (2, M^+^-C_6_H_5_), 321 (2, M^+^-C_6_H_5_N_2_), 295 (2, M^+^-C_8_H_7_N_2_), 267 (7, M^+^-C_8_H_7_N_4_), 242 (12, M^+^-C_12_H_12_N_2_^−^), 217 (5, M^+^-C_14_H_13_N_2_), 191 (5, M^+^-C_15_H_13_N_3_), 190 (10, M^+^-C_14_H_12_N_4_), 164 (9, M^+^-C_15_H_12_N_5_), 114 (35, M^+^-C_21_H_18_N_3_), 88 (25, M^+^-C_22_H_18_N_4_), 76 (71, M^+^-C_21_H_14_N_6_), 62 (17, M^+^-C_23_H_18_N_5_) and 50 (33, M^+^-C_24_H_18_N_5_); Anal. Calc. for C_27_H_18_N_6_ (426.47): C, 76.04; H, 4.25; N, 19.71%, found: C, 75.90; H, 4.15; N, 19.36%.

*2,5-Diphenyl-10-chloroindolo[2,3-e]pyrazolo[1',5':3",4"]pyrimido[2",1"-c][1,2,4]triazines* (**13c**). Yield 94%; m.p. 276–278 °C; IR (cm^−1^): 1645 (indole ring C=N), 1619 (pyrazole ring C=N), 1563 (triazine ring C=N) and 1453 (pyrimidine ring C=C); MS, *m/z* (%): 446 (2, M^+^ ), 369 (1, M^+^-C_6_H_5_), 341 (2, M^+^-C_6_H_5_N_2_), 315 (5, M^+^-C_8_H_7_N_2_), 287 (10, M^+^-C_8_H_7_N_4_), 262 (8, M^+^-C_12_H_12_N_2_^−^), 217 (4, M^+^-C_13_H_10_ClN_2_), 210 (3, M^+^-C_14_H_12_N_4_), 191 (3, M^+^-C_14_H_10_ClN_3_), 184 (2, M^+^-C_15_H_12_N_5_), 114 (4, M^+^-C_20_H_15_ClN_3_), 88 (3, M^+^-C_21_H_15_ClN_4_), 76 (100, M^+^-C_20_H_11_ClN_6_), 62 (3, M^+^-C_22_H_15_ClN_5_) and 50 (44, M^+^-C_23_H_15_ClN_5_); Anal. Calc. for C_26_H_15_ClN_6_ (446.89): C, 69.88; H, 3.38; Cl, 7.93; N, 18.81%, found: C, 69.69; H, 3.16; Cl, 7.70; N, 18.42%.

#### 3.2.9. 4-Bromo-2,5-diphenylindolo[2,3-e]pyrazolo[1',5':3",4"]pyrimido[2",1"-c][1,2,4]triazines **14a–c**

A solution of bromine (0.06 mL, 0.0012 mol) in acetic acid (10 mL) was gradually added to a suspension of **13a–c** (0.001 mol) in acetic acid (10 mL) with stirring for three hours at room temperature. The reaction mixture was then poured onto crushed ice. The products that separated out were filtered off, washed with water, dried and crystallized from dimethylformamide as brown needles.

*4-Bromo-2,5-diphenylindolo[2,3-e]pyrazolo[1',5':3",4"]pyrimido[2",1"-c][1,2,4]triazine* (**14a**). Yield 91%; m.p. 282–284 °C; IR (cm^−1^): 1659 (indole ring C=N), 1616 (pyrazole ring C=N), 1551 (triazine ring C=N) and 1462 (pyrimidine ring C=C); ^1^H-NMR (DMSO-*d_6_*, δ, ppm): 7.26 (d, 1H, aromatic-H), 7.33 (s, 1H, pyrazole-H), 7.36–7.63 (m, 9H, aromatic-H), 8.05 (d, 2H, aromatic-H), 8.20 (d, 2H, aromatic-H); MS, *m/z* (%): 491 (1, M^+^), 307 (1, M^+^-C_7_H_6_BrN), 306 (1, M^+^-C_6_H_5_BrN_2_), 281 (1, M^+^-C_8_H_6_BrN_2_), 253 (1, M^+^-C_8_H_6_BrN_4_), 228 (2, M^+^-C_12_H_11_BrN_2_^−^), 217 (1, M^+^-C_13_H_10_BrN_2_), 191 (1, M^+^-C_14_H_10_BrN_3_), 176 (2, M^+^-C_14_H_11_BrN_4_), 150 (2, M^+^-C_15_H_11_BrN_5_), 114 (2, M^+^-C_20_H_15_BrN_3_), 88 (5, M^+^-C_21_H_15_BrN_4_), 76 (100, M^+^-C_20_H_11_BrN_6_), 62 (5, M^+^-C_22_H_15_BrN_5_) and 50 (33, M^+^-C_23_H_15_BrN_5_); Anal. Calc. for C_26_H_15_BrN_6_ (491.34): C, 63.56; H, 3.08; Br, 16.26; N, 17.10%, found: C, 63.41; H, 2.86; Br, 15.94; N, 16.72%.

*4-Bromo-2,5-diphenyl-10-methylindolo[2,3-e]pyrazolo[1',5':3",4"]pyrimido[2",1"-c][1,2,4]triazine *(**14b**). Yield 92%; m.p. 308–310 °C; IR (cm^−1^): 1674 (indole ring C=N), 1631 (pyrazole ring C=N), 1562 (triazine ring C=N) and 1477 (pyrimidine ring C=C); ^1^H-NMR (DMSO-*d_6_*, δ, ppm): 2.43 (s, 3H, CH_3_), 7.11 (d, 1H, aromatic-H), 7.28 (s, 1H, pyrazole-H), 7.34-7.64 (m, 7H, aromatic-H), 7.92 (d, 2H, aromatic-H), 7.99 (s, 1H, aromatic-H), 8.13 (d, 2H, aromatic-H); MS, *m/z* (%): 505 (8, M^+^ ), 321 (1, M^+^-C_7_H_6_BrN), 320 (5, M^+^-C_6_H_5_BrN_2_), 295 (1, M^+^-C_8_H_6_BrN_2_), 267 (1, M^+^-C_8_H_6_BrN_4_), 242 (1, M^+^-C_12_H_11_BrN_2_^−^), 217 (1, M^+^-C_14_H_12_BrN_2_), 191 (1, M^+^-C_15_H_12_BrN_3_), 190 (6, M^+^-C_14_H_11_BrN_4_), 164 (3, M^+^-C_15_H_11_BrN_5_), 114 (19, M^+^-C_21_H_17_BrN_3_), 88 (17, M^+^-C_22_H_17_BrN_4_), 76 (100, M^+^-C_21_H_13_BrN_6_), 62 (8, M^+^-C_23_H_17_BrN_5_) and 50 (27, M^+^-C_24_H_17_BrN_5_); Anal. Calc. for C_27_H_17_BrN_6_ (505.37): C, 64.17; H, 3.39; Br, 15.81; N, 16.63%, found: C, 64.07; H, 3.24; Br, 15.53; N, 16.29%.

*4-Bromo-2,5-diphenyl-10-chloroindolo[2,3-e]pyrazolo[1',5':3",4"]pyrimido[2",1"-c][1,2,4]triazine* (**14c**). Yield 92%; m.p. 240–242 °C; IR (cm^−1^): 1667 (indole ring C=N), 1616 (pyrazole ring C=N), 1550 (triazine ring C=N) and 1449 (pyrimidine ring C=C); MS, *m/z* (%): 525 (1, M^+^), 341 (2, M^+^-C_7_H_6_BrN), 340 (1, M^+^-C_6_H_5_BrN_2_), 315 (1, M^+^-C_8_H_6_BrN_2_), 287 (13, M^+^-C_8_H_6_BrN_4_), 262 (3, M^+^-C_12_H_11_BrN_2_^−^), 217 (1, M^+^-C_13_H_9_BrClN_2_), 210 (1, M^+^-C_14_H_11_BrN_4_), 191 (1, M^+^-C_14_H_9_BrClN_3_), 184 (2, M^+^-C_15_H_11_BrN_5_), 114 (2, M^+^-C_20_H_14_BrClN_3_), 88 (8, M^+^-C_21_H_14_BrClN_4_), 76 (100, M^+^-C_20_H_10_BrClN_6_), 62 (14, M^+^-C_22_H_14_BrClN_5_) and 50 (59, M^+^-C_23_H_14_BrClN_5_); Anal. Calc. for C_26_H_14_BrClN_6_ (525.79): C, 59.39; H, 2.68; Br, 15.20; Cl, 6.74; N, 15.98%, found: C, 59.16; H, 2.57; Br, 14.73; Cl, 6.34; N, 15.71%.

#### 3.2.10. 3-Bromo-2,5-diphenylindolo[2,3-e]pyrazolo[1',5':3",4"]pyrimido[2",1"-c][1,2,4]triazines **17a–c**

A solution of (*Z*)-**4a–c** (0.0005 mol) in phosphorus oxychloride (5 mL) was heated at 70–80 °C for two hours. The mixture was cooled and poured onto crushed ice and basified with potassium hydrogen carbonate to pH = 9. The products were filtered off, washed with water, dried and crystallized from dimethylformamide.

*3-Bromo-2,5-diphenylindolo[2,3-e]pyrazolo[1',5':3",4"]pyrimido[2",1"-c][1,2,4]triazine* (**17a**). Yield 90%; m.p. 302–304 °C; IR (cm^−1^): 1641 (indole ring C=N), 1619 (pyrazole ring C=N), 1561 (triazine ring C=N) and 1474 (pyrimidine ring C=C); ^1^H-NMR (DMSO-*d_6_*, δ, ppm): 6.93 (d, 1H, aromatic-H), 7.01–7.92 (m, 9H, aromatic-H), 7.94 (s, 1H, pyrimidine-H), 8.05 (d, 2H, aromatic-H), 8.28 (d, 2H, aromatic-H); MS, *m/z* (%): 491 (1, M^+^), 307 (13, M^+^-C_7_H_6_BrN), 306 (1, M^+^-C_6_H_5_BrN_2_), 281 (4, M^+^-C_8_H_6_BrN_2_), 253 (13, M^+^-C_8_H_6_BrN_4_), 228 (18, M^+^-C_12_H_11_BrN_2_^−^), 217 (6, M^+^-C_13_H_10_BrN_2_), 191 (5, M^+^-C_14_H_10_BrN_3_), 176 (5, M^+^-C_14_H_11_BrN_4_), 150 (10, M^+^-C_15_H_11_BrN_5_), 114 (31, M^+^-C_20_H_15_BrN_3_), 88 (2, M^+^-C_21_H_15_BrN_4_), 76 (2, M^+^-C_20_H_11_BrN_6_), 62 (1, M^+^-C_22_H_15_BrN_5_) and 50 (100, M^+^-C_23_H_15_BrN_5_); Anal. Calc. for C_26_H_15_BrN_6_ (491.34): C, 63.56; H, 3.08; Br, 16.26; N, 17.10%, found: C, 63.41; H, 2.92; Br, 15.83; N, 16.79%.

*3-Bromo-2,5-diphenyl-10-methylindolo[2,3-e]pyrazolo[1',5':3",4"]pyrimido[2",1"-c][1,2,4]triazine* (**17b**). Yield 90%; m.p. 200–202 °C; IR (cm^−1^): 1667 (indole ring C=N), 1619 (pyrazole ring C=N), 1567 (triazine ring C=N) and 1454 (pyrimidine ring C=C); ^1^H-NMR (DMSO-*d_6_*, δ, ppm): 2.34 (s, 3H, CH_3_), 7.12 (d, 1H, aromatic-H), 7.19–7.67 (m, 8H, aromatic-H), 7.69 (s, 1H, pyrimidine-H), 8.00 (d, 2H, aromatic-H), 8.09 (d, 2H, aromatic-H); MS, *m/z* (%): 505 (1, M^+^), 321 (1, M^+^-C_7_H_6_BrN), 320 (1, M^+^-C_6_H_5_BrN_2_), 295 (1, M^+^-C_8_H_6_BrN_2_), 267 (1, M^+^-C_8_H_6_BrN_4_), 242 (1, M^+^-C_12_H_11_BrN_2_^−^), 217 (1, M^+^-C_14_H_12_BrN_2_), 191 (1, M^+^-C_15_H_12_BrN_3_), 190 (4, M^+^-C_14_H_11_BrN_4_), 164 (2, M^+^-C_15_H_11_BrN_5_), 114 (30, M^+^-C_21_H_17_BrN_3_), 88 (12, M^+^-C_22_H_17_BrN_4_), 76 (100, M^+^-C_21_H_13_BrN_6_), 62 (19, M^+^-C_23_H_17_BrN_5_) and 50 (69, M^+^-C_24_H_17_BrN_5_); Anal. Calc. for C_27_H_17_BrN_6_ (505.37): C, 64.17; H, 3.39; Br, 15.81; N, 16.63%, found: C, 63.70; H, 3.24; Br, 15.52; N, 16.20%.

*3-Bromo-2,5-diphenyl-10-chloroindolo[2,3-e]pyrazolo[1',5':3",4"]pyrimido[2",1"-c][1,2,4]triazine* (**17c**). Yield 90%; m.p. 248–250 °C; IR (cm^−1^): 1667 (indole ring C=N), 1625 (pyrazole ring C=N), 1564 (triazine ring C=N) and 1456 (pyrimidine ring C=C); MS, *m/z* (%): 525 (2, M^+^), 341 (1, M^+^-C_7_H_6_BrN), 340 (1, M^+^-C_6_H_5_BrN_2_), 315 (1, M^+^-C_8_H_6_BrN_2_), 287 (2, M^+^-C_8_H_6_BrN_4_), 262 (1, M^+^-C_12_H_11_BrN_2_^−^), 217 (3, M^+^-C_13_H_9_BrClN_2_), 210 (2, M^+^-C_14_H_11_BrN_4_), 191 (4, M^+^-C_14_H_9_BrClN_3_), 184 (5, M^+^-C_15_H_11_BrN_5_), 114 (8, M^+^-C_20_H_14_BrClN_3_), 88 (15, M^+^-C_21_H_14_BrClN_4_), 76 (100, M^+^-C_20_H_10_BrClN_6_), 62 (12, M^+^-C_22_H_14_BrClN_5_) and 50 (36, M^+^-C_23_H_14_BrClN_5_); Anal. Calc. for C_26_H_14_BrClN_6_ (525.79): C, 59.39; H, 2.68; Br, 15.20; Cl, 6.74; N, 15.98%, found: C, 59.12; H, 2.49; Br, 14.72; Cl, 6.31; N, 15.54%.

#### 3.2.11. 4-Iodo-2,5-diphenylindolo[2,3-e]pyrazolo[1',5':3",4"]pyrimido[2",1"-c][1,2,4]triazines **15a**,**b**

A solution of iodine monochloride (0.20 g, 0.0012 mol) in acetic acid (10 mL) was gradually added to a suspension of **13a**,**b** (0.001 mol) in acetic acid (10 mL) with stirring for three hours at room temperature. The reaction mixture was then poured onto crushed ice and the products that separated out were filtered off, washed with water, dried and crystallized from dimethylformamide.

*4-Iodo-2,5-diphenylindolo[2,3-e]pyrazolo[1',5':3",4"]pyrimido[2",1"-c][1,2,4]triazine* (**15a**). Yield 90%; m.p. 314–316 °C; IR (cm^−1^): 1652 (indole ring C=N), 1620 (pyrazole ring C=N), 1567 (triazine ring C=N) and 1438 (pyrimidine ring C=C); ^1^H-NMR (DMSO-*d_6_*, δ, ppm): 7.22 (d, 1H, aromatic-H), 7.37 (t, 1H, aromatic-H), 7.41 (s, 1H, pyrazole-H), 7.42-7.59 (m, 8H, aromatic-H), 8.16 (d, 2H, aromatic-H), 8.20 (d, 2H, aromatic-H); MS, *m/z* (%): 538 (1, M^+^), 307 (3, M^+^-C_7_H_6_IN), 306 (2, M^+^-C_6_H_5_IN_2_), 281 (2, M^+^-C_8_H_6_IN_2_), 253 (1, M^+^-C_8_H_6_IN_4_), 228 (1, M^+^-C_12_H_11_IN_2_^−^), 217 (1, M^+^-C_13_H_10_IN_2_), 191 (1, M^+^-C_14_H_10_IN_3_), 176 (1, M^+^-C_14_H_11_IN_4_), 150 (1, M^+^-C_15_H_11_IN_5_), 114 (4, M^+^-C_20_H_15_IN_3_), 88 (4, M^+^-C_21_H_15_IN_4_), 76 (100, M^+^-C_20_H_11_IN_6_), 62 (7, M^+^-C_22_H_15_IN_5_) and 50 (29, M^+^-C_23_H_15_IN_5_); Anal. Calc. for C_26_H_15_IN_6_ (538.34): C, 58.01; H, 2.81; I, 23.57; N, 15.61%, found: C, 57.82; H, 2.66; I, 23.19; N, 15.14%.

*4-Iodo-2,5-diphenyl-10-methylindolo[2,3-e]pyrazolo[1',5':3",4"]pyrimido[2",1"-c][1,2,4]triazine* (**15b**). Yield 89%; m.p. 298–300 °C; IR (cm^−1^): 1689 (indole ring C=N), 1622 (pyrazole ring C=N), 1560 (triazine ring C=N) and 1449 (pyrimidine ring C=C); ^1^H-NMR (DMSO-*d_6_*, δ, ppm): 2.31 (s, 3H, CH_3_), 6.83 (d, 1H, aromatic-H), 7.47 (s, 1H, pyrazole-H), 7.09–7.58 (m, 8H, aromatic-H), 8.00 (d, 2H, aromatic-H), 8.25 (d, 2H, aromatic-H); MS, *m/z* (%): 552 (1, M^+^), 321 (1, M^+^-C_7_H_6_IN), 320 (1, M^+^-C_6_H_5_IN_2_), 295 (1, M^+^-C_8_H_6_IN_2_), 267 (1, M^+^-C_8_H_6_IN_4_), 242 (2, M^+^-C_12_H_11_IN_2_^−^), 217 (1, M^+^-C_14_H_12_IN_2_), 191 (1, M^+^-C_15_H_12_IN_3_), 190 (1, M^+^-C_14_H_11_IN_4_), 164 (2, M^+^-C_15_H_11_IN_5_), 114 (8, M^+^-C_21_H_17_IN_3_), 88 (4, M^+^-C_22_H_17_IN_4_), 76 (100, M^+^-C_21_H_13_IN_6_), 62 (12, M^+^-C_23_H_17_IN_5_) and 50 (37, M^+^-C_24_H_17_IN_5_); Anal. Calc. for C_27_H_17_IN_6_ (552.37): C, 58.71; H, 3.10; I, 22.97; N, 15.21%, found: C, 58.58; H, 2.89; I, 22.62; N, 14.94%.

#### 3.2.12. 4-Nitro-2,5-diphenyl-10-methylindolo[2,3-e]pyrazolo[1',5':3",4"]pyrimido[2",1"-c][1,2,4] triazines **16b**,**c**

A mixture of nitric acid (d 1.14, 1 mL) and sulfuric acid (d 1.84, 1 mL) in glacial acetic acid (10 mL) was added gradually to a suspension of **13b**,**c** (0.001 mol) in acetic acid (10 mL) with stirring for three hours at room temperature. The reaction mixture was then poured onto crushed ice and the products that separated out were filtered off, washed with water, dried and crystallized from dimethylformamide.

*4-Nitro-2,5-diphenyl-10-methylindolo[2,3-e]pyrazolo[1',5':3",4"]pyrimido[2",1"-c][1,2,4]triazine* (**16b**). Yield 92%; m.p. 312–314 °C; IR (cm^−1^): 1650 (indole ring C=N), 1617 (pyrazole ring C=N), 1555 (triazine ring C=N), 1449 (pyrimidine ring C=C) and 1421, 1391 (NO_)_; MS, *m/z* (%): 471 (2, M^+^), 321 (2, M^+^-C_7_H_6_N_2_O_2_), 320 (2, M^+^-C_6_H_5_N_3_O_2_), 295 (2, M^+^-C_8_H_6_N_3_O_2_), 267 (2, M^+^-C_8_H_6_N_5_O_2_), 242 (2, M^+^-C_12_H_11_N_3_O_2_^−^), 217 (3, M^+^-C_14_H_12_N_3_O_2_), 191 (3, M^+^-C_15_H_12_N_4_O_2_), 190 (2, M^+^-C_14_H_11_N_5_O_2_), 164 (3, M^+^-C_15_H_11_N_6_O_2_), 114 (3, M^+^-C_21_H_17_N_4_O_2_), 88 (4, M^+^-C_22_H_17_N_5_O_2_), 76 (70, M^+^-C_21_H_13_N_7_O_2_), 62 (4, M^+^-C_23_H_17_N_6_O_2_) and 50 (100, M^+^-C_24_H_17_N_6_O_2_); Anal. Calc. for C_27_H_17_N_7_O_2_ (471.47): C, 68.78; H, 3.63; N, 20.80%, found: C, 68.49; H, 3.52; N, 20.49%.

*4-Nitro-2,5-diphenyl-10-chloroindolo[2,3-e]pyrazolo[1',5':3",4"]pyrimido[2",1"-c][1,2,4]triazine* (**16c**). Yield 92%; m.p. 226–228 °C; IR (cm^−1^): 1681 (indole ring C=N), 1622 (pyrazole ring C=N), 1530 (triazine ring C=N), 1442 (pyrimidine ring C=C), and 1426,1342 (NO_2_); MS, *m/z* (%): 491 (1, M^+^), 341 (1, M^+^-C_7_H_6_N_2_O_2_), 340 (1, M^+^-C_6_H_5_N_3_O_2_), 315 (4, M^+^-C_8_H_6_N_3_O_2_), 287 (2, M^+^-C_8_H_6_N_5_O_2_), 262 (2, M^+^-C_12_H_11_N_3_O_2_^−^), 217 (2, M^+^-C_13_H_9_ClN_3_O_2_), 210 (2, M^+^-C_14_H_11_N_5_O_2_), 191 (3, M^+^-C_14_H_9_ClN_4_O_2_), 184 (1, M^+^-C_15_H_11_N_6_O_2_), 114 (9, M^+^-C_20_H_14_ClN_4_O_2_), 88 (4, M^+^-C_21_H_14_ClN_5_O_2_), 76 (100, M^+^-C_20_H_10_ClN_7_O_2_), 62 (8, M^+^-C_22_H_14_ClN_6_O_2_) and 50 (30, M^+^-C_23_H_14_ClN_6_O_2_); Anal. Calc. for C_26_H_14_ClN_7_O_2_ (491.89): C, 63.49; H, 2.87; Cl, 7.21; N, 19.93%, found: C, 63.27; H, 2.71; Cl, 6.83; N, 19.54%.

### 3.3. Biological Screening: Antibacterial Activity Tests

The antibacterial activities of compounds **3–7** and **13–16** were tested against three Gram-positive (*Bacillus subtilis*,*Micrococcus luteus*, and *Staphylococcus aureus*) and two Gram-negative (*Escherichia coli*, and *Pseudomonas aeruginosa) *clinical multidrug resistant (MDR) test bacteria isolated from diabetic foot ulcers. Used clinical bacteria are with MIC > 256 µg/mL for amino- glycosides, penicillins, 1st–3rd generations of cephalosparins and ciprofloxacin and ofloxacin fluoro quinolines.

Bioactivities (Minimum Inhibitory Concentration, MIC) were determined according to the recommendations of NCCLS [36] and Massoud *et al.* [37].

All compounds were first dissolved in DMSO and serially diluted to have final concentrations from 256–1 µg/mL culture medium at 1.5 dilution factor. The MIC value of a compound is the lowest concentration that inhibits the bacterial growth. The smaller the MIC value the more active is the compound. Compounds with MIC values above 256 µg/mL are considered to be inactive. It should be taken into consideration, before discussing the bioactivity of this set of compounds, that the used bacteria, being MDR, are highly resistant to the antibiotics of choice that are commonly used to treat infections by these bacteria.

From the data presented in Table 1, it is clear that from the 28 tested compounds, twelve compounds were active, six active against *B. subtilis*, four active against *M. luteus*, two active against *S. aureus*, none active against *E. coli* and three active against *Ps. aeruginosa*. (*E*)-**3b**, **7a** and **14a** were active against two of the tested bacteria and other were active against only one. This means that none of the tested compounds have broad antibacterial spectrum except (*E*)-**3b**.

**Table 1:** Minimum inhibitory concentration (MIC) (µg/mL) of compounds **3–7** and **13–16** against selected bacterial strains.

Compound No.	Gram-positive			Gram-negative	
	*B. subtilis*	*M. luteus*	*S. aureus*	*E. coli*	*Ps. aeruginosa*
	MIC (µg/mL)				
(*E*)-**3a**	>256	>256	>256	>256	>256
(*E*)-**3b**	>256	**24**	>256	>256	**48**
(*E*)-**3c**	>256	>256	>256	>256	>256
(*Z*)-**3a**	>256	>256	>256	>256	>256
(*Z*)-**3b**	>256	**32**	>256	>256	>256
(*Z*)-**3c**	>256	>256	>256	>256	>256
(*Z*)-**4a**	>256	>256	>256	>256	>256
(*Z*)-**4b**	>256	>256	>256	>256	**16**
(*Z*)-**4c**	>256	>256	>256	>256	>256
(*Z*)-**5a**	>256	>256	>256	>256	>256
(*Z*)-**5b**	>256	>256	>256	>256	>256
(*Z*)-**5c**	**12**	>256	>256	>256	>256
(*Z*)-**6a**	>256	>256	>256	>256	>256
(*Z*)-**6b**	>256	>256	>256	>256	>256
(*Z*)-**6c**	>256	>256	>256	>256	**24**
(*Z*)-**7a**	>256	**32**	**48**	>256	>256
(*Z*)-**7b**	**24**	>256	>256	>256	>256
(*Z*)-**7c**	>256	>256	>256	>256	>256
**13a**	>256	>256	>256	>256	>256
**13b**	**16**	>256	>256	>256	>256
**13c**	>256	>256	>256	>256	>256
**14a**	>256	**24**	**32**	>256	>256
**14b**	>256	>256	>256	>256	>256
**14c**	**32**	>256	>256	>256	>256
**15a**	**32**	>256	>256	>256	>256
**15b**	>256	>256	>256	>256	>256
**16b**	**48**	>256	>256	>256	>256
**16c**	>256	>256	>256	>256	>256

The previous results showed clearly the structure activity relationships. Thus, the presence of methyl group at position-5 of the indolinone ring (*E* and *Z*)-**3b** generates antibacterial activity. Also, the presence of electron attracting group (Br, I, NO_2_ and C_6_H_5_N_2_) at position-3 or position-4** 4b**, **5c**,** 6c**,** 7a**,** 7b**,** 14a**,** 14c**,** 15a** and **16b** produces antibacterial activities.

## 4. Conclusions

In conclusion, the two geometrical isomers (*E* and *Z*)-3-{2-(2,5-diphenylpyrazolo[1,5-*c*]pyrimidin-7-yl)hydrazono}indolin-2-ones and their substituted derivatives have been synthesized. The target compounds 2,5-diphenylindolo[2,3-*e*]pyrazolo[1',5':3",4"]pyrimido[2",1"-*c*][1,2,4]-triazines were achieved by dehydrative cyclisation of pyrazolopyrimidinoindolinonehydrazones and their reactivity towards electrophilic substitution reactions were also studied. Some of the synthesized compounds were found to possess slight to moderate activity against the microorganisms *Bacillus subtilis*, *Micrococcus luteus*, *Staphylococcus aureus*, *Escherichia coli * and *Pseudomonas aeruginosa*.
